# Effect of Denture Base Stiffness on Stress Redistribution in an Idealized Complete-Denture Model Under Static Bilateral Clenching: A Finite Element Analysis

**DOI:** 10.3390/ma19183961

**Published:** 2026-09-18

**Authors:** Jeong-Hee Seo, Jeong-Hyeon Lee, Won-Gi Kim, Sung-Jae Lee, Bongju Kim, Dong-Wook Han

**Affiliations:** 1 Research and Development Center, DENTIS Co., Ltd., Daegu 41065, Republic of Korea; sjh00@dentis.co.kr; 2 National Institute of Food and Drug Safety Evaluation, Ministry of Food and Drug Safety, Osong 28159, Republic of Korea; jeonghyeon6089@gmail.com; 3 Department of Dental Technology, Daegu Health College, Daegu 41453, Republic of Korea; wgkim@dhc.ac.kr; 4 Department of Biomedical Engineering, Inje University, Gimhae 50834, Republic of Korea; biomech100@gmail.com; 5 Dental Life Science Research Institute, Seoul National University Dental Hospital, Seoul 03080, Republic of Korea; 6 Department of Cogno-Mechatronics Engineering, Pusan National University, Busan 46241, Republic of Korea; 7 School of Transdisciplinary Engineering, University College, Pusan National University, Busan 46241, Republic of Korea

**Keywords:** denture-base stiffness, complete denture, finite element analysis, static bilateral clenching, stress redistribution

## Abstract

Denture base elastic-property assignment may influence how mechanical stress is distributed between a complete denture and its supporting tissues. This study compared rigid and flexible denture base material conditions in an idealized maxillomandibular finite element (FE) model at the final static bilateral clenching step. The model represented a fully edentulous geometry with coincident denture–gingiva surfaces connected by a tie constraint. The model did not include an initial interface gap, interface separation or sliding, extraction sockets, tissue remodeling, or time-dependent misfit. Geometry, mesh, boundary conditions, and loading were identical between the two conditions, whereas both the Young’s modulus and Poisson’s ratio assigned to the denture base differed. The peak magnitude of the minimum principal stress in the gingiva and the model-predicted local peak von Mises stress in the denture base elements were evaluated. Under the adopted mesh configuration, the flexible property assignment was associated with reductions of 28.6% and 35.3% in gingival compressive stress magnitude and increases of 70.5% and 73.9% in local denture base peak stress in the maxilla and mandible, respectively. These local peak differences are exploratory, model-dependent observations and have not been demonstrated to be mesh-independent. The findings do not establish clinical adaptation, contact pressure, tissue protection, deformation, durability, or performance in immediate dentures.

## 1. Introduction

Immediate dentures are inserted following tooth extraction to provide transitional esthetic and functional rehabilitation during healing [1,2,3,4,5]. Post-extraction changes in the residual ridge and mucosa can alter prosthesis adaptation [6,7]. Such geometric changes may, in turn, modify the mechanical load path within the denture–supporting tissue system. These clinical considerations provide a general motivation for investigating denture base mechanics. Direct simulation of an immediate-denture condition, however, would require post-extraction socket geometry, evolving tissue morphology, loss of adaptation, and a removable-denture contact formulation.

Polymethyl methacrylate has traditionally been used for denture bases because of its esthetics, processability, and cost-effectiveness [8,9]. Digital manufacturing, including CAD/CAM and 3D printing, can reduce manual processing steps, preserve design data, and facilitate remanufacture [10,11]. The mechanical properties of 3D-printed dental and denture base polymers nevertheless depend on the resin system, build orientation, post-processing, and aging conditions [12,13,14,15,16].

Denture base material assignments can differ in Young’s modulus, Poisson’s ratio, deformation capacity, and strength [17,18]. A flexible material-property assignment may alter load transfer within the denture–gingiva system compared with a rigid reference assignment. This comparison can be examined computationally by maintaining the same geometry, mesh, boundary conditions, and applied load while changing the assigned denture base elastic-property set. Such an analysis represents a controlled comparison between two specified property assignments rather than an isolation of the independent effect of Young’s modulus or a direct prediction of clinical denture performance.

The incremental contribution of the present study was to apply a newly characterized flexible resin elastic-property assignment to the previously developed maxillomandibular model and to compare its model-specific stress pattern with that of the inherited rigid reference assignment while keeping geometry, mesh, boundary conditions, and loading unchanged. Therefore, this study aimed to compare gingival and denture base stress distributions between these two assigned material-property conditions at the final static bilateral clenching step. It was hypothesized that the flexible assignment, defined by a lower Young’s modulus and a different Poisson’s ratio, would be associated with lower calculated compressive stress in the gingiva and higher local stress within the denture base. The immediate-denture context was retained only as clinical motivation and was not represented as a post-extraction computational condition.

## 2. Materials and Methods

**Figure 1:**
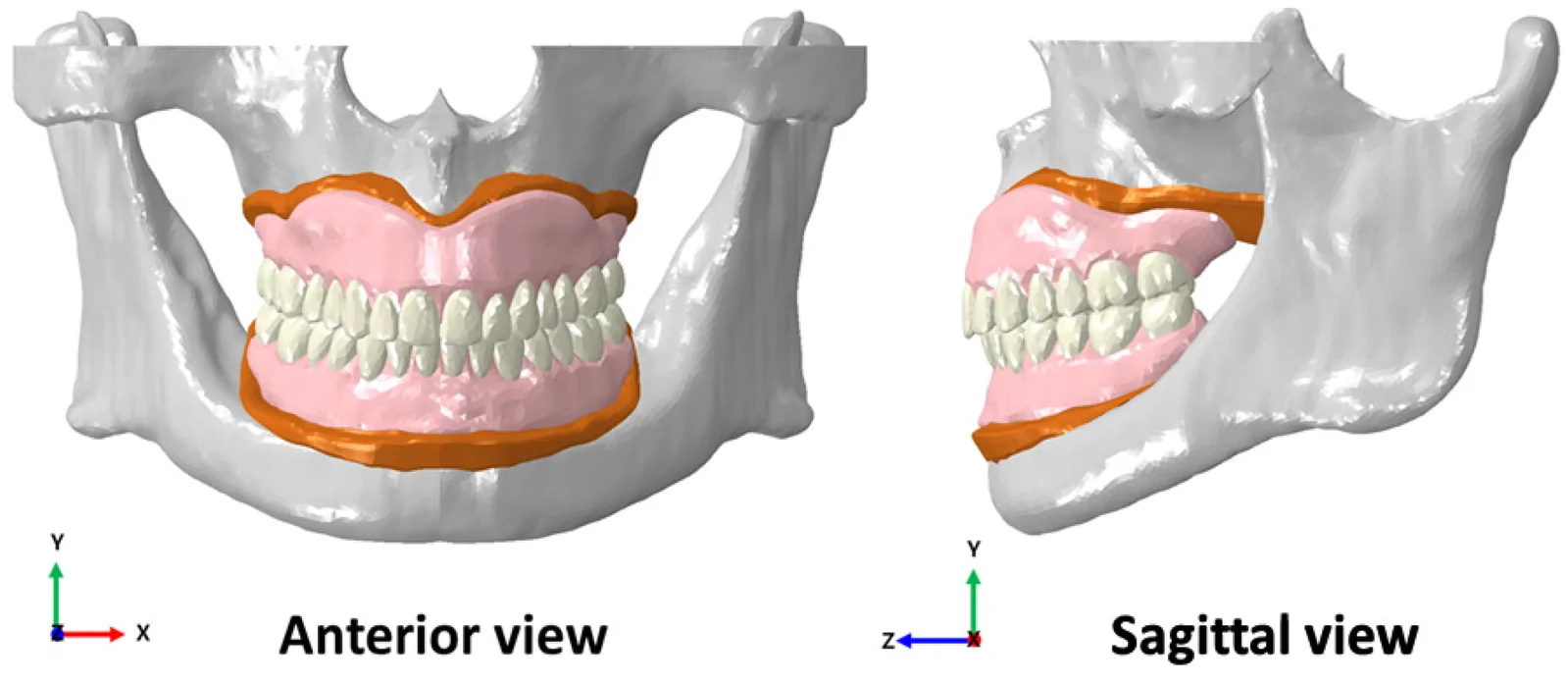
Idealized maxillomandibular FE model with maxillary and mandibular complete dentures.

A previously developed maxillomandibular FE model was used [19]. The model included the maxilla, mandible, gingiva, and maxillary and mandibular complete-denture bases (Figure 1). The denture geometry was designed using dental CAD software (EDS v1.1, DENTIS Co., Ltd., Daegu, Republic of Korea). The denture and gingival surfaces were geometrically coincident and connected using a tie constraint, producing a zero-gap interface that did not permit separation or sliding. Extraction sockets, postoperative edema, post-extraction tissue remodeling, progressive loss of adaptation, and time-dependent material behavior were not represented. Accordingly, the model was used as an idealized complete-denture system for a controlled comparison of denture base material-property assignments and not as a direct simulation of a clinical immediate denture. The rigid condition was assigned a Young’s modulus of 4500 MPa and a Poisson’s ratio of 0.35, as adopted from the parent model [19,20]. The flexible condition was based on ZMD-1000B Flexible Pink resin (DENTIS Co., Ltd.) manufactured using a ZENITH L2 dental photopolymerization printer (DENTIS Co., Ltd.). The material-property values were supplied by the manufacturer’s materials-development team from testing commissioned to an independent testing laboratory. Type I dog-bone specimens were prepared in accordance with ASTM D638-14, and Poisson’s ratio testing was commissioned in accordance with ASTM E132-17 [21,22]. The specimens were printed at a 45° on-edge build orientation with a 50 μm layer thickness, washed in 95% ethanol for 5 min, and post-cured for 15 min using a 405 nm LED in a ZENITH Cure Solution unit (DENTIS Co., Ltd.) at a nominal 70% power setting (0.7 W, with 1 W defined as 100%). The post-cured specimens were shipped to the testing laboratory and tested within 1 week. Six specimens were tested at a crosshead speed of 5 mm/min. The supplied Young’s modulus was 257 ± 13.8 MPa, and 257 MPa was assigned as an effective isotropic input. The supplied Poisson’s ratio was 0.40; specimen-level variability and the underlying axial/transverse strain records were not available to the authors. The available summary did not specify the strain-measurement instrumentation, fitting interval, or conditioning and testing environment. Because the two conditions were not characterized using matched manufacturing and testing protocols and both Young’s modulus and Poisson’s ratio differed, the analysis was interpreted as a comparison of two assigned elastic-property sets rather than a direct product-to-product comparison or an isolated Young’s modulus effect.

A limited global structural validation of the flexible complete-denture condition was conducted using the same static compression method described for the complete-denture model in the parent work and the associated master’s thesis [19,20]. Six complete-denture specimens were positioned in a universal testing machine (MTS 858, MTS Systems Corp., Eden Prairie, MN, USA) using a test fixture corresponding to the FE setup and were compressed under displacement control at 5 mm/min. In the corresponding FE simulation, the fixture and denture were arranged to reproduce the experimental configuration, and loads were applied in 200 N increments up to 1400 N. Stiffness was calculated from the load–displacement response. The experimental and FE stiffness values differed by less than 5%. This comparison was used only as a limited check of global load–displacement behavior; it did not validate the local gingival or tooth–denture base stress field.

All components were modeled as homogeneous, isotropic, linearly elastic materials to permit a controlled comparison under identical geometry and loading conditions. Comparable homogeneous, isotropic material idealizations have been used in dental prosthesis FE studies [23,24]. The flexible material assignment used the Young’s modulus and Poisson’s ratio supplied from testing, but did not reproduce build-direction dependence [14] or nonlinear deformation, viscoelasticity, creep, plasticity, and time-dependent behavior [25]. The calculated values were therefore interpreted as model-specific differences between the two assigned elastic-property sets rather than as the independent effect of either material constant or as absolute predictions of clinical tissue or material behavior. The material properties assigned to the FE components are summarized in Table 1.

**Figure 2:**
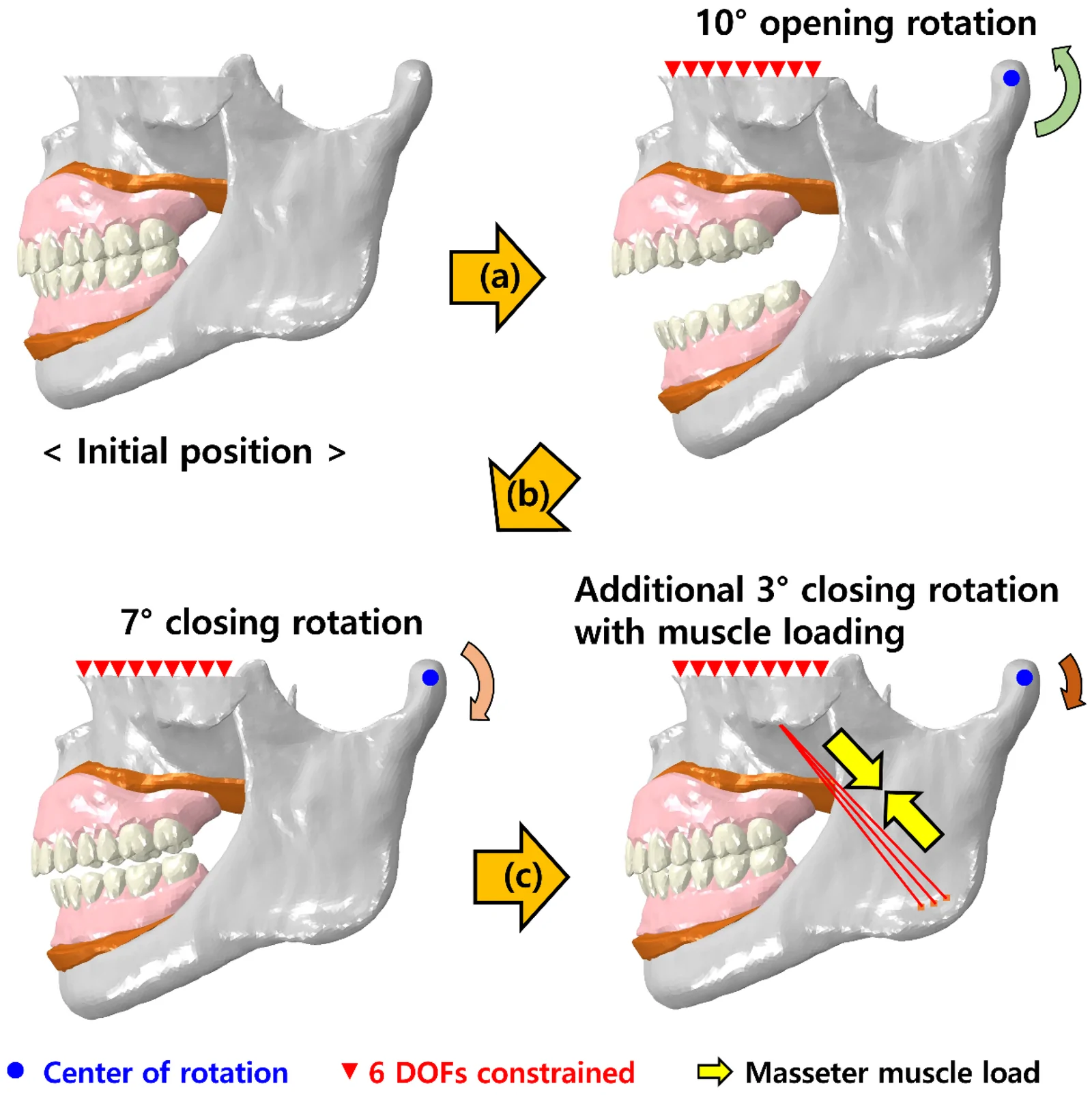
Prescribed mandibular sequence and boundary conditions: (**a**) 10° opening rotation; (**b**) 7° closing rotation; and (**c**) an additional 3° closing rotation maintained during application of approximately 340 N per side (680 N bilaterally) through the masseter truss elements.

The prescribed mandibular sequence followed the framework of the parent model [19] and consisted of a 10° opening rotation in Step 1, a 7° closing rotation in Step 2, and an additional 3° closing rotation in Step 3. The prescribed rotation remained enforced during Step 3 while the masseter loads were applied. Three truss elements were used on each side, and a connector-force value of −113.3 N was assigned to each element according to its local force direction, producing approximately 340 N per side and 680 N bilaterally. This loading condition was retained as a standardized reference from the parent model and was not intended to represent a patient-specific bite force or a loading value specific to immediate-denture users. Only the masseter muscles were represented. The opening and closing steps were used to reach the final configuration, whereas the reported stress outcomes were extracted only at the final static bilateral clenching step (Figure 2).

Rigid beam elements connected the mandibular condylar regions to a central reference point defining the rotational axis. Translational degrees of freedom at the reference point were constrained. Sagittal-plane rotation was the permitted rotational degree of freedom and was controlled by the prescribed rotation in each step; the Step 3 rotation remained enforced during application of the muscle forces. The superior surface of the maxillary alveolar bone was fixed in all six degrees of freedom. The maxilla, mandible, gingiva, denture bases, artificial teeth, and occlusal block were discretized using C3D4 4-node linear tetrahedron elements. The condylar connectors and muscle components were represented using B31 2-node linear beam elements and T3D2 2-node linear truss elements, respectively. The rigid and flexible conditions used identical geometry, connectivity, mesh topology, and boundary conditions and contained 226,850 nodes and 1,035,935 elements. The cortical–cancellous bone and cortical bone–gingiva interfaces were constructed using shared nodes, forming continuous meshes with distinct material properties assigned to the respective regions. Tie constraints were applied only at the gingiva–denture base and denture base–artificial tooth interfaces, where separation and sliding were prevented. Surface-to-surface interactions were limited to the maxillary and mandibular anterior teeth, including the canines; the maxillary posterior teeth and superior surfaces of the occlusal blocks; and the mandibular posterior teeth and inferior surfaces of the occlusal blocks. These interactions used surface-to-surface discretization with a small-sliding formulation. Tangential behavior was the only explicitly assigned contact property and was represented using a penalty friction formulation with a coefficient of 0.2. No separate user-defined normal contact behavior, hard contact option, normal penalty-enforcement option, or normal contact stiffness was specified. No initial penetration was present. Contact stabilization was applied through the Abaqus/Standard contact controls using a stabilization coefficient of 0.02.

All analyses were performed using ABAQUS/CAE 2017 (Dassault Systèmes SE, Vélizy-Villacoublay, France) with the Abaqus/Standard solver and the Static, General procedure. Geometric nonlinearity was disabled (NLGEOM=NO). The 1.2 mm characteristic mesh configuration was adopted from the convergence assessment of the parent model [19], and identical mesh topology was used for the two material conditions. No targeted local mesh refinement assessment was performed for the gingival or tooth–denture base peaks, and no regional or percentile-based stress metric was prospectively defined. Consequently, neither the absolute local peak values nor their relative differences were demonstrated to be mesh-independent. Stress outcomes were extracted at the final static bilateral clenching step. Stress values were reviewed at the Element Nodal output position in Abaqus/CAE. For gingival compression, the S, Min. Principal invariant was displayed, and the most negative element-nodal value was reported in the text as an absolute compressive stress magnitude. For the denture base, the S, Mises invariant was displayed for surface elements adjacent to the tooth–denture base interface and at the intaglio surface. The displayed values used nodal averaging with a 75% averaging threshold. The authors did not independently average the underlying stress components or recalculate the invariants outside Abaqus/CAE. The same output position, invariant display, and averaging settings were used for both material conditions. No separate averaging threshold, contact stabilization, material-property, or other parametric sensitivity analysis was performed. Because the simulations were deterministic, the comparisons were descriptive and no inferential statistical tests were performed.

## 3. Results

The rigid and flexible material conditions were compared using identical geometry, mesh, boundary conditions, and loading. The evaluated outcomes were the peak magnitude of the minimum principal stress in the gingiva and the model-predicted local peak von Mises stress in the denture base elements at the final static bilateral clenching step.

### 3.1. Compressive Principal Stress in the Gingiva

**Figure 3:**
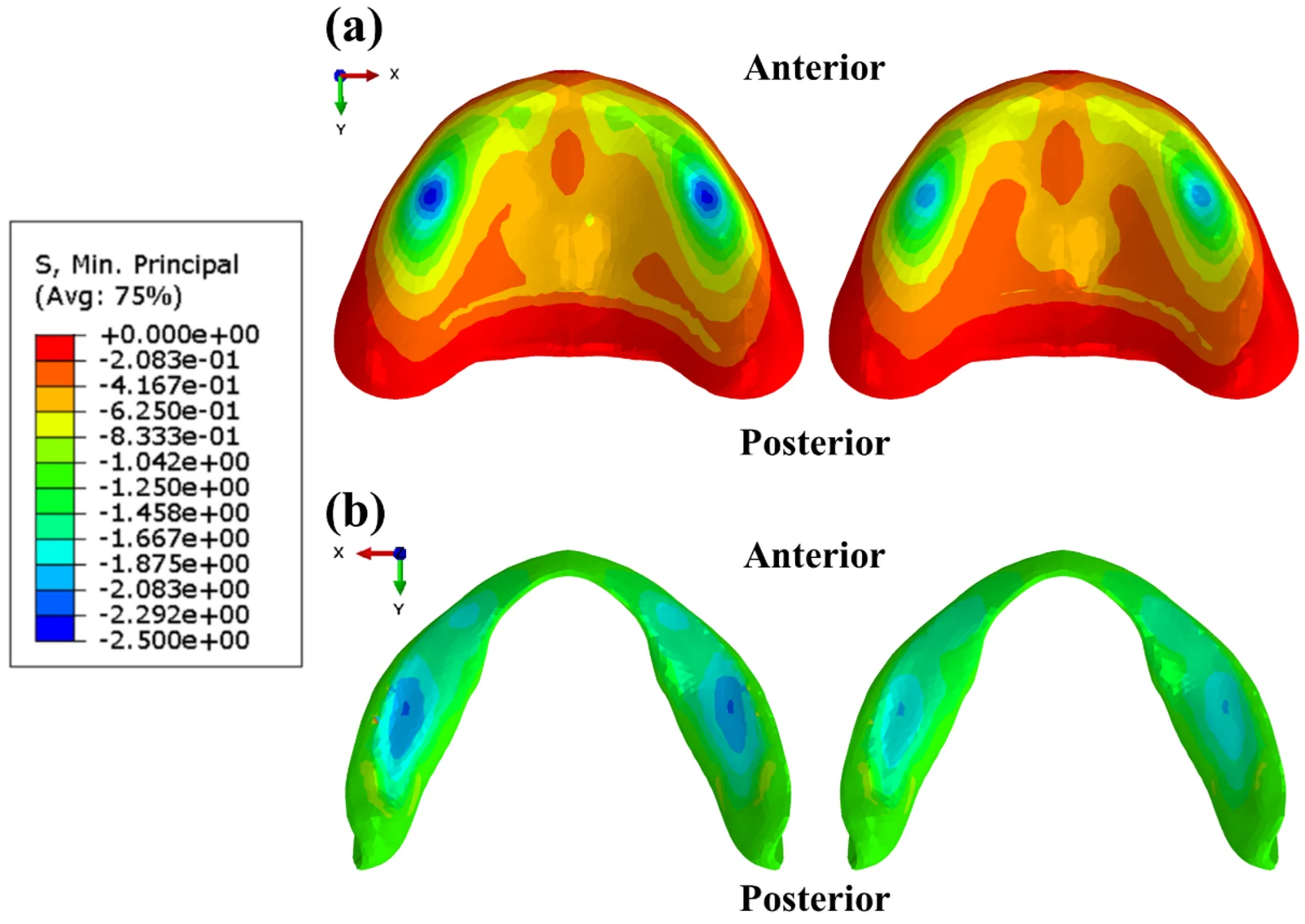
Distribution of the element-nodal minimum principal stress (SP1) in the gingiva at the final static bilateral clenching step: (**a**) maxilla with the rigid condition on the left and flexible condition on the right; and (**b**) mandible with the rigid condition on the left and flexible condition on the right. Negative values indicate compression, whereas the peak values reported in the text are also expressed as absolute magnitudes for comparison. The reported peak regions were located bilaterally between the maxillary second premolar and first molar and in the mandibular first molar region. Identical contour limits were used for the rigid and flexible conditions. Contours are displayed on the deformed geometry using a deformation scale factor of 1.0.

The most negative element-nodal minimum principal stress values were −2.1 MPa in the maxillary gingiva and −1.7 MPa in the mandibular gingiva for the rigid condition. The corresponding values for the flexible condition were −1.5 MPa and −1.1 MPa. Expressed as absolute compressive stress magnitudes, these values represented descriptive reductions of 28.6% and 35.3%, respectively, under the adopted mesh configuration. In both conditions, the reported peak regions were located bilaterally between the maxillary second premolar and first molar and in the mandibular first molar region (Figure 3).

### 3.2. Peak Von Mises Stress in the Denture Base

**Figure 4:**
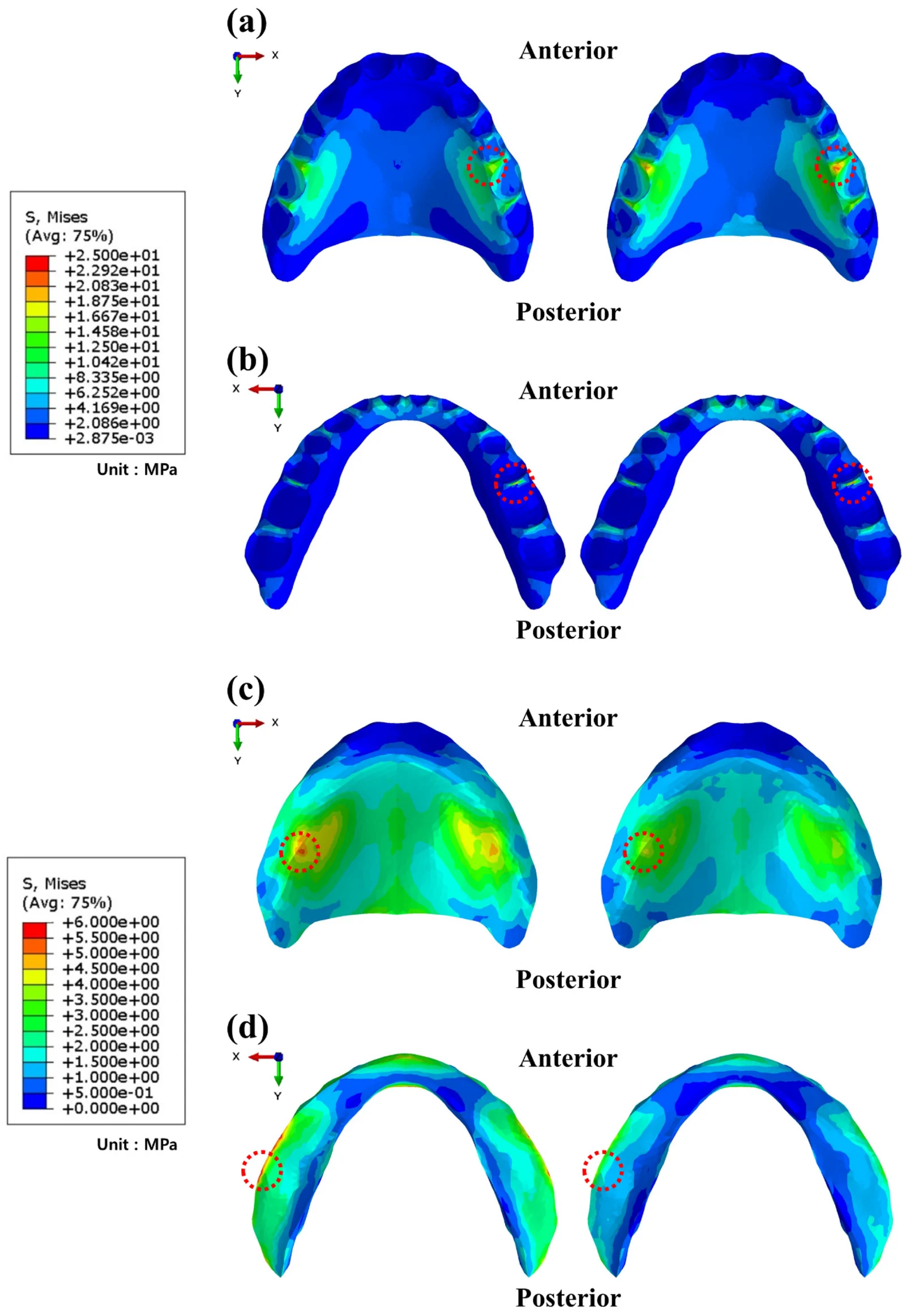
Von Mises stress distribution in the denture base surface elements at the final static bilateral clenching step. Local stress adjacent to the tooth–denture base interface is shown for (**a**) the maxilla and (**b**) the mandible. Intaglio-surface stress is shown for (**c**) the maxilla and (**d**) the mandible. In each pair, the rigid condition is shown on the left and the flexible condition on the right. Dashed circles indicate the locations of the reported maximum values, including the 24.9 MPa maxillary and 19.3 MPa mandibular values for the flexible condition; all reported maxima occurred on the displayed surfaces. Stress values in the legends are expressed in MPa. Identical contour limits were used for the rigid and flexible conditions. Contours are displayed on the deformed geometry using a deformation scale factor of 1.0.

Exploratory, model-predicted local peak von Mises stress was evaluated at the Element Nodal output position in surface elements adjacent to the tooth–denture base interface and at the intaglio surface. All reported maxima occurred on the displayed surfaces. Under the adopted mesh configuration, the local peak adjacent to the tooth–denture base interface occurred between the maxillary second premolar and first molar and in the mandibular first molar region. The rigid condition produced local peak values of 14.6 MPa in the maxilla and 11.1 MPa in the mandible. The corresponding values for the flexible condition were 24.9 MPa and 19.3 MPa, representing descriptive increases of 70.5% and 73.9%, respectively. At the corresponding intaglio-surface regions, the rigid condition produced values of 5.7 MPa in the maxilla and 5.1 MPa in the mandible, whereas the flexible condition produced values of 4.3 MPa and 4.2 MPa, representing descriptive reductions of 24.6% and 17.6%, respectively (Figure 4). These values are mesh- and model-dependent local observations and should not be interpreted as mesh-independent, regionally averaged, or direct failure measures.

The model-predicted stress outcomes for the two material conditions are summarized in Table 2.

## 4. Discussion

Under the adopted 1.2 mm mesh configuration, comparison of the two assigned denture base elastic-property sets showed a model-specific difference in calculated stress distribution within an otherwise identical FE model. The flexible assignment was associated with lower peak compressive stress magnitude in the gingiva and higher exploratory local peak von Mises stress in surface elements adjacent to the tooth–denture base interface. These observations describe the outputs of the specified model and post-processing settings; they do not establish mesh-independent peak magnitudes or a general material law.

Both Young’s modulus and Poisson’s ratio differed between the two denture base assignments. The flexible assignment combined a lower Young’s modulus (257 MPa versus 4500 MPa) with a different Poisson’s ratio (0.40 versus 0.35) and was associated with a different structural response within the tied continuum model. Because no constant-Poisson’s ratio or material-property sensitivity analysis was performed, the present comparison cannot isolate the independent contribution of either parameter. The observed pattern is therefore described as a difference between the two specified elastic-property assignments rather than as an effect caused solely by Young’s modulus.

The gingival outcome represents element-nodal minimum principal stress within a continuum connected to the denture through a tie constraint. It is not equivalent to physiological denture–mucosa contact pressure because the interface did not permit opening or sliding. Similarly, the reported denture base values are exploratory surface local peaks and may be influenced by local geometry, mesh density, element formulation, and nodal averaging. The use of identical geometry, mesh topology, loading, output position, and post-processing settings permits an internally controlled descriptive comparison, but does not establish that the absolute or relative local peaks are insensitive to discretization. Targeted local refinement and a prospectively defined regional or percentile-based stress measure are needed to verify the numerical robustness of these observations.

Denture displacement, base flexure, strain, retention, border adaptation, occlusal stability, occlusal reaction force, fatigue, and tooth–base debonding were not evaluated as outcomes. A separate diagnostic assessment of displacement, strain, reaction force, averaging threshold, or stabilization sensitivity was also not performed. Accordingly, the present stress outputs cannot determine whether the flexible assignment would produce clinically acceptable deformation, whether the applied muscle force input corresponds to a particular transmitted occlusal force, or whether the reported peaks are insensitive to the stabilization and averaging settings. The local von Mises stress values are retained only as exploratory comparative elastic outputs and are not used to predict yielding, fracture, fatigue life, or clinical durability.

Several limitations should be acknowledged. The analysis used a single fully edentulous geometry with a zero-gap tied denture–gingiva interface. Extraction sockets, postoperative edema, post-extraction remodeling, progressive misfit, separation, sliding, and physiological contact pressure were not modeled. All materials were represented as homogeneous, isotropic, and linearly elastic, and geometric nonlinearity was disabled. The flexible property assignment was supplied from third-party testing of six Type I specimens printed at a single orientation; the underlying strain records, Poisson’s ratio variability, and detailed conditioning and testing environment were not available to the authors. The rigid and flexible conditions were not characterized using matched manufacturing protocols, and both Young’s modulus and Poisson’s ratio differed. Only the masseter muscles and one standardized bilateral loading condition were included. The compression comparison supported only global load–displacement behavior and did not validate local stress. No targeted local mesh-convergence analysis, regional stress metric, displacement or strain analysis, occlusal reaction-force analysis, averaging or stabilization diagnostic, or parametric sensitivity analysis was performed. The reported peak values and between-condition differences are therefore exploratory observations restricted to the adopted mesh, material assignments, boundary conditions, and post-processing settings.

## 5. Conclusions

Under the adopted mesh configuration, zero-gap tied-interface assumption, and final static bilateral clenching condition, the comparison showed a model-specific pattern of lower calculated gingival compressive stress magnitude and higher exploratory local denture base stress for the flexible elastic-property assignment. The local peak magnitudes and their between-condition differences require verification through targeted mesh refinement and a predefined regional stress measure before they can be considered numerically robust. These results do not establish clinical adaptation, tissue protection, contact pressure, deformation, yielding, durability, or performance in immediate dentures.

## Figures and Tables

**Table 1:** Material properties assigned to the finite element model.

Component		Elastic Modulus (MPa)	Poisson’s Ratio	Ref.
Alveolar bone	Cortical bone	18,000	0.31	[19]
	Cancellous bone	500	0.30	[19]
Gingiva		3	0.3	[19]
Artificial teeth		3000	0.35	[19]
Occlusal block		4	0.45	[19]
Denture base	Rigid	4500	0.35	[19]
	Flexible	257	0.40	Present study

**Table 2:** Summary of model-predicted stress outcomes at the final static bilateral clenching step.

Outcome	Arch	Rigid Condition	Flexible Condition	Relative Change
Gingival compressive stress magnitude (MPa)	Maxilla	2.1	1.5	−28.6%
	Mandible	1.7	1.1	−35.3%
PVMS adjacent to the tooth–base interface (MPa)	Maxilla	14.6	24.9	+70.5%
	Mandible	11.1	19.3	+73.9%
Intaglio-surface PVMS (MPa)	Maxilla	5.7	4.3	−24.6%
	Mandible	5.1	4.2	−17.6%

## Data Availability

The original contributions presented in this study are included in the article. Further inquiries can be directed to the corresponding authors.
